# Evaluating DNA Function Understanding in Genomic Language Models Using Evolutionarily Implausible Sequences

**DOI:** 10.1021/acssynbio.6c00024

**Published:** 2026-06-09

**Authors:** Shiyu Jiang, Xuyin Liu, Zitong Jerry Wang

**Affiliations:** † Center for Interdisciplinary Studies, School of Science, 3463 Westlake University, Hangzhou 310030, China

**Keywords:** genomic language model, synthetic biology, mutation prediction, gene expression, regulatory genomics, mechanistic understanding

## Abstract

Genomic language models (gLMs) hold promise for generating novel, functional DNA sequences for synthetic biology. A critical challenge is determining whether gLMs understand sequence function or merely memorize training patterns derived from natural genomes. We introduce Nullsettes, an evaluation framework that measures how well models predict *in silico* loss-of-function (LOF) mutations in synthetic expression cassettes lacking evolutionary precedent. Across state-of-the-art gLMs, we find a consistent failure to detect strong LOF mutations. Predictive accuracy declines sharply when the original nonmutant has lower model likelihood, indicating reliance on evolutionary pattern-matching rather than mechanistic understanding of gene expression. These results expose core limitations in how gLMs generalize to engineered genetic constructs, and emphasize the need for evaluation and modeling strategies that explicitly test for functional understanding.

## Graphical abstract

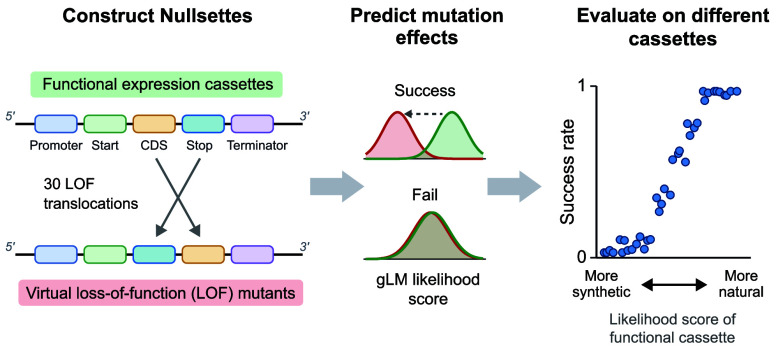

## 1 Background

Genomic language models (gLMs) are increasingly used for DNA sequence design in synthetic biology, where they learn a probability distribution over naturally occurring DNA sequences that approximate evolutionary plausibility.^1,2^ This plausibility reflects factors such as the presence of common regulatory motifs or similarity to known natural genomes. For example, gLMs tend to assign higher likelihoods to promoters with frequent natural motifs than to random synthetic promoters.

Evolutionary plausibility can be a useful proxy for biological function, as gLMs have already demonstrated strong zero-shot performance in predicting effects of genetic mutations. In genomic language models, higher model likelihoods can correlate with improved functional properties. For example, gLM likelihoods enable genome-wide prediction of variant effects^3^ and 5′ untranslated region (UTR) optimization.^4^ However, it remains unclear whether gLMs have a true understanding of sequence function or simply recall evolutionarily similar sequences at prediction time.

For synthetic biology applications, design objectives such as high expression, orthogonality, or tight regulatory control often come at a fitness cost, making highly effective constructs evolutionarily implausible.^5^ Therapeutic and industrial applications therefore require functional understanding beyond evolutionary plausibility. Yet current evaluations of gLMs largely focus on endogenous or endogenous-derived regulatory and splicing contexts,^6^ leaving open the question of how well models generalize to non-natural constructs. Many high-value challenges involve sequences with little or no evolutionary precedent, including synthetic promoters that drive expression beyond natural limits,^7^ therapeutic vectors incorporating synthetic regulatory elements, and metabolic pathways for small-molecule production.^8^

Meeting these challenges requires evaluation frameworks that test whether gLMs can generalize beyond evolutionary patterns to capture functional principles, the mechanistic rules that ultimately determine whether a sequence works.

Fundamental mechanisms of gene expression are conserved across all domains of life, making synthetic expression cassettes an ideal test of functional understanding. We introduce Nullsettes, a framework for evaluating whether gLMs can predict loss-of-function (LOF) mutations in synthetic expression cassettes. We create these LOF mutations *in silico* by rearranging key regulatory elements, such as promoters and start codons, within nonmutant cassettes. These rearrangements disrupt the canonical sequence structure required for transcription and translation, rendering the sequences nonfunctional. We curate nonmutant cassettes from massively parallel reporter assay (MPRA) data sets.^9–12^ GLMs assign low likelihood to these cassettes as they contain genetic elements from evolutionarily distant species, and incorporate random sequences functioning as promoters.

We find that most state-of-the-art gLMs perform poorly on zero-shot LOF prediction, particularly under contexts that deviate strongly from natural sequence statistics. For example, model performance worsens across nearly all 14 gLMs when cassettes include functional promoters drawn from random sequence libraries, or when mutations cause more severe functional disruption. Notably, all models show a sharp drop in prediction accuracy as the likelihood of the nonmutant sequence decreases, indicating that gLMs rely heavily on pattern-matching to their evolutionary priors rather than on functional understanding of gene expression. Accurate predictions only emerge when the nonmutant likelihood surpasses a particular threshold. Differences in performance across models show that functional generalization may depend more on the relevance and quality of pretraining data than on the sheer size of the data set. We further benchmarked AlphaGenome,^13^ a recently published supervised sequence-to-expression model, on a transcriptional-disruption subset of Nullsettes, showing its comparative performance among top-performing gLMs. These findings highlight fundamental limitations in how current gLMs generalize to engineered constructs, and underscore the need for evaluation frameworks and modeling strategies that prioritize the functional understanding of DNA sequences.

## 2 Results

We design Nullsettes as a framework to test whether genomic language models (gLMs) understand functional gene expression in sequences that lack evolutionary plausibility. Nullsettes consists of zero-shot mutation effect prediction tasks, where synthetic expression cassettes are subjected to virtual loss-of-function (LOF) mutations created by rearranging key genetic elements.

In a canonical eukaryotic expression cassette (Figure 1A, i–ii), control elements including the promoter, start codon, coding sequence (CDS), stop codon, and terminator must appear in a specific 5′-3′ order to enable proper transcription and translation. Rearranging these elements, for example, placing a terminator upstream of the CDS (iii) or positioning the CDS downstream of the stop codon (iv), disrupts this structure and results in nonfunctional gene expression. Nullsettes focuses on single-element translocations that eliminate functional protein expression and cannot be rescued by any upstream or downstream sequences (Figure 1B). From these criteria, we obtain 11 mutation types for eukaryotic and 19 for prokaryotic cassettes, with additional prokaryotic variants involving rearrangement of ribosome binding site (RBS) (Supplementary Tables S1 and S2).

**Figure 1:**
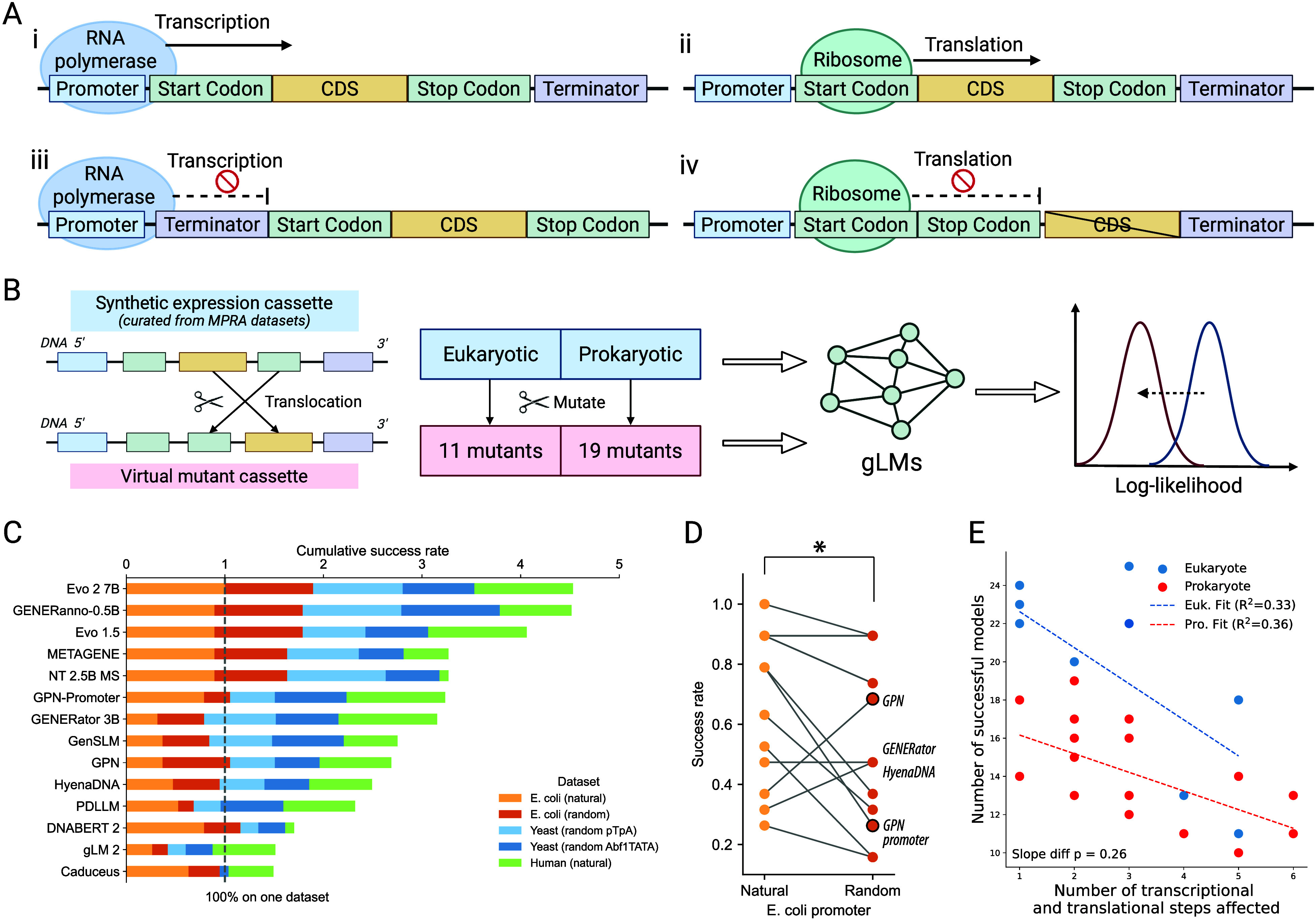
Overview of Nullsettes. A) Schematic examples showing transcription (i) and translation (ii) becoming impaired when the order of control element such as terminator (iii) or stop codon (iv) is altered. B) Nullsettes consists of 11 and 19 mutant variants for each eukaryotic and prokaryotic expression cassette, respectively, by translocating control elements, and evaluates how well gLMs can predict these loss-of-function mutations based on changes in log-likelihood. We curate functional nonmutant cassettes from five MPRA data sets. C) Cumulative success rates of representative gLMs evaluated on five MPRA data sets, with promoter either being random sequences or based on natural promoter (indicated in brackets). The two yeast data sets use promoters containing both natural motifs (Abf1-TATA, polyT-polyA) and random sequences. Dashed lines denote 100% success on one data set. Models are sorted by cumulative success rate. D) Success rates of models in identifying Nullsettes mutants for *E. coli* expression cassettes with either naturally derived or random promoters. Each line connects the performance of a single model across the two sets of cassettes. Statistical significance with adjusted p-value <0.05 for a one-sided paired permutation test. E) Scatter plot depicting the relationship between the number of transcriptional and translational steps affected (*x*-axis) and the number of models that successfully assign low pseudolikelihood (*y*-axis) across Nullsettes mutants. Successful detection is defined as a statistically significant reduction in LL relative to the nonmutant (see Methods). Each point represents a distinct type of Nullsettes mutant, with eukaryotic (*n* = 11) and prokaryotic (*n* = 19) variants shown in blue and red, respectively. Dashed lines indicate linear regression fits for each group. ANCOVA test (*p* = 0.26) difference in slope between two groups.

To ensure these tasks test generalization beyond evolutionary prior, we generate virtual mutants from synthetic expression cassettes with low gLM-assigned likelihoods but strong gene expression in either *E. coli*, yeast, or human, drawn from five massively parallel reporter assay (MPRA) data sets^9–12^ (Figure 1B). These cassettes combine sequences from distantly related species, for example, GFP as CDS from *Aequorea victoria* with promoters and terminators from bacteria, human, or mouse. Many cassettes even use random (but functional) promoters, which further lower gLM-assigned likelihood (Supplementary Figure S1). Together, these cassettes enable evaluation of model generalization to functional yet evolutionarily implausible sequences across species.

Given all cassettes in a data set, we evaluate mutation effect prediction in a zero-shot setting by comparing the distribution of nonmutant log-likelihood (LL) to the distribution of corresponding Nullsettes mutants (Figure 1B). We consider a model capable of identifying a mutation type if the mutant LL is significantly lower than the nonmutant LL, as determined by a paired permutation test. A model’s success rate on the data set is the proportion of mutation types identified.

Across five data sets, we found that most genomic language models perform poorly in predicting LOF mutants in Nullsettes. We evaluated 14 representative models (and 35 variants; Supplementary Tables S3–S5) spanning state-of-the-art approaches to genomic language modeling, with 11 out of 14 models achieving <50% success rate on at least one data set, failing to identify strong LOF mutations (Figure 1C). Only Evo2-7B and GENERanno-0.5B deliver strong, consistent performance (Supplementary Figure S2), with mean LL shifts of 3–4 standard deviations away from the nonmutant distribution for successful identifications (Supplementary Figure S3). Failed predictions were generally not accompanied by greater uncertainty in mean LL differences, as measured by bootstrapped 95% confidence-interval widths (Supplementary Figure S4), suggesting that gLMs do not reliably distinguish incorrect predictions. We also evaluated AlphaGenome,^13^ a supervised sequence-to-expression model, which performed competitively on transcription-disrupting mutants, second only to GENERanno (Supplementary Figure S5). A full comparison was not possible because AlphaGenome predicts only transcription-level readouts and cannot be assessed on translation-disrupting mutants.

GENERanno matched Evo2–7B performance despite having 14-fold fewer parameters and using 12-fold less pretraining data, potentially due to its curated pretraining on actively expressed regions of the genome.^14^ Since our evaluation scores sequences involved in gene expression, GENERanno’s likelihoods are plausibly better calibrated for this task than those of models pretrained on the whole genome, suggesting that task-relevant data curation can yield substantial gains in functional generalization when pretraining data is limited. A similar pattern has been observed in protein language modeling, where more curated pretraining data sets can outperform larger but less curated ones for variant effect prediction.^15^ Supporting this hypothesis, performance did not consistently improve with scaling across four model families (Supplementary Figure S6).

Current gLMs recognize what natural expression cassettes look like, but not how they work, failing to generalize to contexts that deviate strongly from natural sequence statistics. Across most models, performance drops significantly when identifying mutants with strong promoters drawn from random sequence libraries compared to promoters derived from nature (*p* = 0.04) (Figure 1D). Models struggle to predict mutation effects when evolutionarily plausibility is not a useful prior for function, as these random sequences are assigned significantly lower LL (Supplementary Figure S1). Exceptions include HyenaDNA, GPN, and GENERator, which maintain performance on random promoters. For GPN, this robustness may reflect its more diverse pretraining across entire genomes, compared to GPN-Promoter which was pretrained exclusively on TSS-adjacent sequences and shows a strong performance drop.

A similar failure emerges across Nullsette mutations, with model performance worsening as mutations disrupt more steps of the gene expression process (Figure 1E). If models understood how genetic elements mechanistically drive expression, their predictions should remain robust or even improve as mutations become more disruptive. Instead, performance declines (linear fit R^2^ = 0.33 and 0.36 for eukaryote and prokaryote respectively), suggesting that models do not reason about functional mechanism, and behave sensibly only within the distribution of natural sequences. We quantify disruption by counting the transcribed and translated elements missing in each mutant. When only one step is affected (e.g., a premature stop codon), the mutant remains close to natural sequence space, and models can often detect the resulting loss of function. But when multiple steps are disrupted (e.g., loss of both transcription and translation), the sequence falls further outside the distribution seen during training, and model outputs become unreliable. Taken together, these results show that current gLMs do not understand regulatory logic, generalizing based on sequence similarity to evolutionary prior.

Across all gLMs, zero-shot performance in mutation effect prediction declines sharply as the log-likelihood (LL) of the original (nonmutant) sequence decreases, reinforcing that models rely on their evolutionary priors rather than a mechanistic understanding of DNA function. If a model understood how mutations disrupt gene expression, its predictions should depend on what function the mutation alters, not on how similar the sequence looks to the model’s training corpus. In that case, LOF mutants would be correctly identified regardless of the LL of the nonmutant cassette. However, we observe a strong dependence between nonmutant LL and success rate. Figure 2A shows this relationship for Evo2-7B and NT-2.5B-MS. For both models, the ability to detect LOF mutations improves consistently as the LL of the nonmutant increases. For example, in the *E. coli* (random) data set,^9^ NT-2.5B-MS fails almost completely when LL < −27, but performs well above 80% when LL > −22. This dependency is not specific to a single model. As shown in Figure 2B, the same correlation between nonmutant LL and prediction accuracy holds across nearly all models, regardless of architecture or training corpus, indicating a shared reliance on sequence prior rather than reasoning about expression mechanisms.

**Figure 2:**
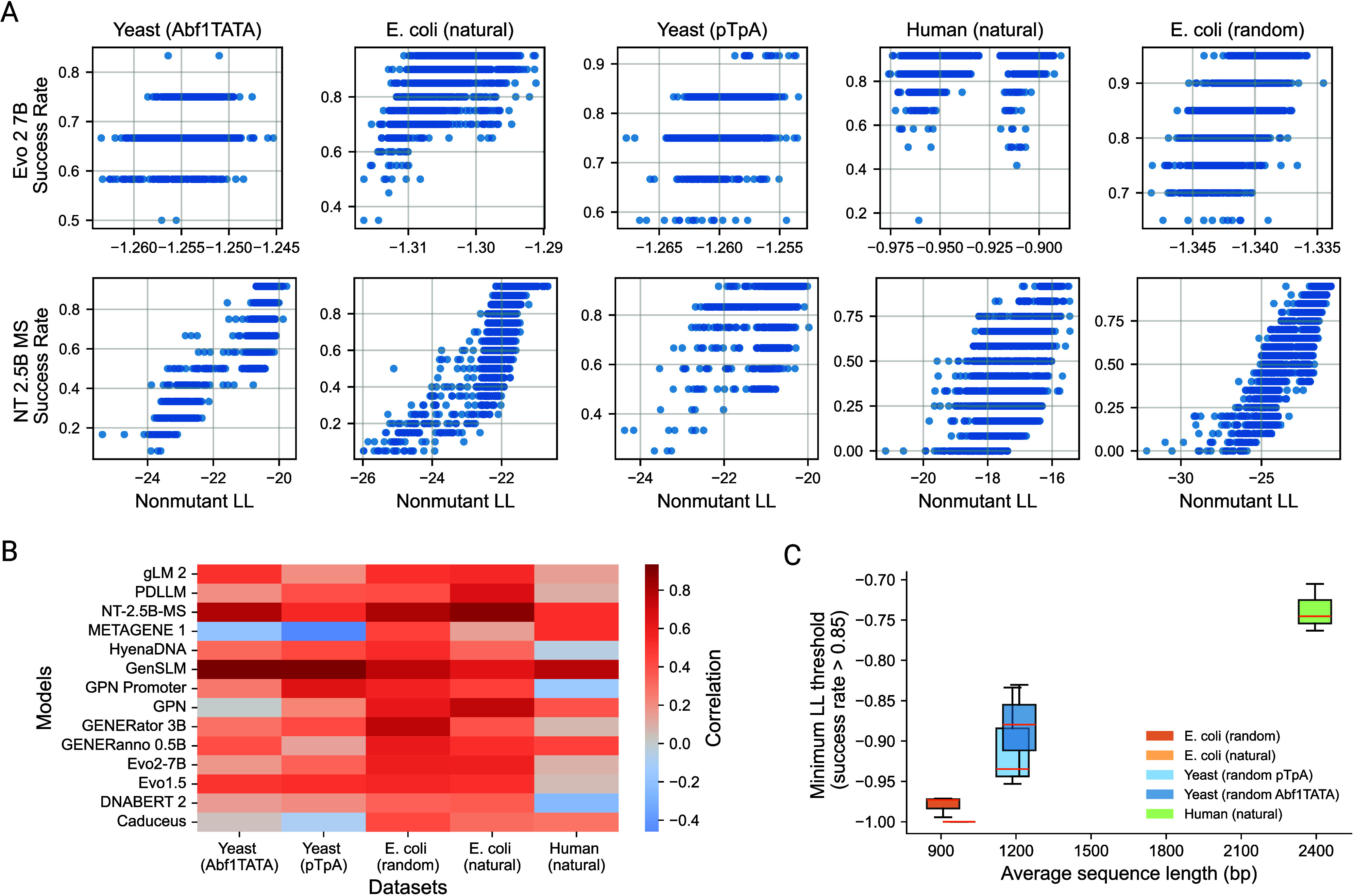
Relationship between gLM likelihood and zero-shot performance on mutation effect prediction. A) Each point represents an expression cassette. Scatterplots show the Nullsettes prediction performance of Evo2–7B and NT-2.5B-MS for cassettes from five data sets. B) Heatmap showing correlation between the gLM log likelihood (LL) of a sequence and gLM success rate on predicting its corresponding Nullsettes, across five data sets and 14 models. C) Log-likelihood (LL) threshold to achieve a success rate >0.85 across data sets of increasing sequence length. Boxplots show the distribution of LL thresholds for top performing models (GENERanno, Evo2–7B, and Evo1.5) across five data sets, rescaled by lowest threshold. Data sets are ordered according to their average sequence length.

This LL-accuracy relationship is not explained by variations in sequence composition or length. Cassette GC content and sequence entropy show little to no correlation with prediction accuracy across data set-model pairs (mean correlations of −0.027 and −0.057, respectively; Supplementary Figure S7). Similarly, the LL-performance correlation persists when we restrict the analysis to cassettes of a fixed length (Supplementary Figure S7), indicating that length does not confound the relationship either.

Among top-performing models, the threshold LL required for high prediction accuracy is consistent and increases with sequence length. Figure 2A shows that the minimum LL threshold for accurate prediction can vary widely across data sets. For example, NT-2.5B-MS achieves a success rate of 0.85 at a threshold LL of approximately −20 for Yeast (Abf1TATA) cassettes, but around −16 for Human (natural). For each model–data set pair, we estimate the LL threshold corresponding to a success rate of 0.85 using linear regression. This success rate was selected as the most stringent criterion achievable across top models: it permits at most one detection error among the prokaryote mutants and two among the eukaryote mutants, while avoiding extrapolation beyond observed performance ranges. Figure 2C reveals that these thresholds are consistent among top performing models and scale with sequence length (after rescaling by the lowest threshold), suggesting that sequence length is an important determinant of the effective LL threshold for zero-shot mutation effect prediction. This relationship persists for different success rate thresholds (Supplementary Figure S8).

## 3 Discussion

In this study, we introduce Nullsettes, a framework for evaluating the ability of genomic language models (gLMs) to understand DNA function by predicting loss-of-function (LOF) mutations in synthetic expression cassettes. Our analysis of 14 state-of-the-art models reveals that most struggle to consistently identify these LOF mutations, often relying on surface-level similarity rather than true functional understanding of regulatory elements. Reinforcing this fact, we show that LOF prediction accuracy declines sharply when nonmutant sequences have low gLM-assigned log-likelihood, as is the case for synthetic sequences. Comparing performance between models, we find a key avenue for improving functional understanding may be prioritizing curated, functionally relevant pretraining data over brute-force scaling.

A limitation of Nullsettes is that performance may depend on how far the tested constructs lie from the training distributions of each model. Although the cassettes follow a standard gene-expression architecture, individual sequence elements may be underrepresented in natural genomic data, which could reduce model performance independently of mechanistic understanding. A separate limitation is that Nullsettes is designed to probe mechanistic generalization rather than as a comprehensive measure of model utility. Models that fail this benchmark may still be useful in settings where natural evolutionary patterns are sufficient, such as optimizing promoters similar to those represented in the training data. Thus, Nullsettes should be viewed as a targeted benchmark for identifying models likely to generalize to synthetic constructs, rather than as a comprehensive measure of model utility.

Our findings complement recent work by Tang et al.,^6^ who evaluated gLM representational power on functional prediction tasks using natural human and mouse genomic sequences. Whereas Tang et al. ask whether gLMs are useful for functional prediction, Nullsettes asks how they make such predictions. This difference in objective motivates a different evaluation design: while Tang et al. focus on natural genomic fragments, Nullsettes uses synthetic but functional expression cassettes spanning a broad range of evolutionary plausibility, thereby revealing how prediction accuracy depends on nonmutant likelihood. This dependence is particularly important for synthetic biology, where high-performing constructs are often evolutionarily unprecedented yet fully functional. Nullsettes shows that many gLMs break down on such constructs, highlighting the need for evaluation frameworks and modeling strategies that prioritize functional understanding for cell engineering.

## 4 Methods

### 4.1 Functional Cassette Curation

#### 4.1.1 Prokaryote

To evaluate genomic language models on the regulatory grammar of prokaryotes, we utilized two data sets derived from synthetic expression constructs in *Escherichia coli*. The first, the Kosuri data set,^11^ comprises combinatorial assemblies of promoters and ribosome binding sites (RBS) upstream of a superfolder GFP (sfGFP) reporter, designed to dissect transcriptional and translational contributions to gene expression. The second, the Lagator data set,^9^ features a large-scale library of random promoter sequences with experimentally measured activity, enabling evaluation of models’ ability to generalize to highly diverse, out-of-distribution regulatory inputs.

#### 4.1.2 Eukaryote

To evaluate the capacity of genomic language models to generalize to eukaryotic regulatory logic, we utilized two large-scale massively parallel reporter assay (MPRA) data sets. The Zahm data set^12^ comprises a library of 6,144 synthetic promoters constructed by combining transcriptional response elements (TREs) from 229 human and mouse transcription factors with minimal promoters. These constructs were assayed across multiple human cell lines and stimulatory conditions to quantify dynamic, stimulus-specific gene expression. The deBoer data set^10^ includes a comprehensive collection of 100 million randomized promoter sequences in yeast, enabling high-resolution dissection of cis-regulatory grammar under an out-of-distribution scenario.

#### 4.1.3 Promoter Selection

To construct decoupled expression cassettes, we selected 1,500 promoters or promoter–RBS pairs from five distinct data sets.

For the Kosuri data set,^11^ we selected candidate promoter–RBS pairs with protein expression levels exceeding μ + 1.5σ (10,165) across the full distribution. In contrast, given the limited number of highly active constructs in the Lagator data set, we directly selected the top 1,500 promoter sequences ranked by protein output. We then compared the log-likelihood (LL) distributions of promoter–RBS groups using genomic language models including Evo1–8k, GENERator-3B, GPN, and NT-2.5B-MS. As the Lagator data set^9^ consists of randomly generated promoters, whereas the Kosuri library is more rationally designed, we prioritized Kosuri promoter–RBS pairs whose LL distributions surpassed those from Lagator (see Supplementary Figure S1), yielding 1,500 expression cassettes from each data set.

deBoer data set^10^ contains two promoter libraries: Abf1TATA and pTpA. Abf1TATA is designed by embedding conserved transcription factor binding sites such as Abf1 and a canonical TATA box, mimicking features of natural yeast promoters. In addition, pTpA consists of synthetic promoters constructed with a poly-T–poly-A architecture. Poly­(dA-dT) sequences are naturally occurring motifs that associate with the centers of nucleosome-free regions in yeast promoters and serve as a hybrid natural + randomized library. We selectively constructed 1,500 active promoters for each data set.

The Zahm data set^12^ comprises synthetic promoters built by coupling one of three minimal promoters—minCMV, minProm, or minTK—with diverse transcriptional response element (TRE) units. TRE units contain Transcription factor binding motifs (TFBMs) mostly derived from human genomes. To construct a representative set of mammalian expression cassettes, we selected the top 1,500 promoter-TRE combinations exhibiting the highest transcriptional activity.

### 4.2 Nullsettes Virtual Mutant Construction

To assess whether genomic language models can detect violations of canonical gene regulatory architecture, we constructed a set of completely nonfunctional expression cassettes called Nullsettes. Nullsettes were generated by systematic component translocations that disrupt the canonical transcription–translation structure while retaining all original components.

We first defined the canonical regulatory architecture for both prokaryote and eukaryote:

- **Prokaryotic systems** (e.g., *E. coli*) contain six ordered elements: Promoter - RBS - Start codon - CDS - Stop codon - Terminator.
- **Eukaryotic systems** (e.g., yeast, mammalian) consist of five ordered elements, lacking an RBS as translation initiation is guided by the 5 ′ cap and the Kozak consensus sequence rather than a defined RBS: Promoter - Start codon - CDS - Stop codon - Terminator.

For each system, we generated all possible single-component translocation mutants, in which one element was relocated to a different position while preserving the identity and total count of elements.

To determine whether a mutant retained functional syntax, we evaluated each sequence against a minimal set of biologically grounded ordering constraints. These constraints ensure that proper CDS expression cannot be rescued by any flanking sequences outside the cassette. Specifically, we consider all circular permutations of each ordering and retain a mutant as a Nullsette only if no circular permutation of the mutant satisfies all three of the following rules:

- **Rule 1: Proper initiation ordering**—Ensures that transcription starts upstream of translation initiation and that the CDS is translated from an upstream start codon. Eukaryotic: Position­(Promoter) < Position­(Start codon) < Position­(CDS) Prokaryotic: Position­(Promoter) < Position­(RBS) < Position­(Start codon) < Position­(CDS)
- **Rule 2: Valid translation termination**—The stop codon must follow the CDS or precede the start codon in circular order. (i) Position­(CDS) < Position­(Stop codon) or, (ii) Position­(Stop codon) < Position­(Start codon)
- **Rule 3: Valid transcription termination**—The terminator must lie downstream of the CDS or upstream of the promoter in circular form. (i) Position­(CDS) < Position­(Terminator) or (ii) Position­(Terminator) < Position­(Promoter)

After applying these rules across all circular permutations, we identified 19 nonfunctional translocation mutants in the prokaryotic system and 11 in the eukaryotic system, all of which violated the defined minimal functional syntax. These mutants are synthetically designed to be completely nonfunctional, challenging models to detect biologically catastrophic yet structurally subtle disruptions in regulatory element ordering.

All mutant sequences are listed in Supplementary Table S1 (prokaryote) and Supplementary Table S2 (eukaryote). Detailed expression cassette architecture of each data set is in Supplementary Table S6.

### 4.3 Baseline Models

We benchmarked a diverse set of 14 self-supervised genomic foundation models that represent current state-of-the-art approaches to DNA language modeling. In general, the models can be categorized based on their tokenization schemes (fixed-length k-mers, single-nucleotide tokens, byte-pair encoding, and hybrid schemes), pretraining strategies (masked language modeling^16^ vs causal language modeling^17^), training corpus diversity (human references, plant genomes, multispecies genomes, and large-scale metagenomic assemblies), and architectural paradigms (CNN-based models, Transformer-based models,^2^ and emerging architectures like StripedHyena^18,19^ and Mamba^20^). Detailed model specifications are concluded in Supplementary Table S4 for causal language modeling-based models and S5 for masked-language-modeling-based models. Given the fundamental differences in the training objectives of these two model classes, sequence scores were computed using model-specific procedures. For MLM-based models, sequence scores were computed from token-level likelihood produced by the masked-language-model head, whereas for CLM-based models, scores were computed autoregressively from next-token logits.

We also included AlphaGenome,^13^ the most recently published sequence-to-expression model, in our comparison. It models regulatory sequence activity by predicting base-resolution functional genomic signals from input DNA sequence. For inference, because AlphaGenome directly predicts RNA-seq signal tracks, we summarized its output by averaging the predicted values across positions corresponding to the coding sequence (CDS) of each cassette, thereby obtaining a single scalar score for each sequence. Nullsettes that disrupt transcription are thus expected to receive lower predicted expression. AlphaGenome is only assessed on mutants that disrupt transcriptional activity, since it predicts only transcription-related readouts and therefore cannot be assessed on mutants that disrupt only translation (around 60% of the benchmark).

### 4.4 Evaluation Metrics

We computed the length-normalized sequence-level log-likelihood (LL) as the sequence-level LL score, where the normalization was performed at either the nucleotide or token level according to each model’s tokenization scheme. To account for the distinct pretraining objectives of causal language models and masked language models, we applied different LL computation strategies.

Specifically, for causal language models (CLM), the log-likelihood of a nucleic acid sequence X = (*x*_1_, *x*_2_, ..., *x*_*n*_) is computed using logits output from the model. Since CLM operates in an autoregressive manner, each token *x*_*t*_ is predicted based on all previous tokens *x* < *t*. Given the model’s *logits*_*t*_ at each position *t*, the probability of the ground-truth token is obtained via softmax:

1 $$P\left(x_{t}|x_{<t}\right)=\frac{\exp\left({\mathrm{logits}}_{t,x_{t}}\right)}{\underset{v\in V}{\sum }\exp\left({\mathrm{logits}}_{t,v}\right)}$$

where *V* represents the vocabulary. The mean log-likelihood is then computed as

2 $${\mathrm{LL}}_{\mathrm{CLM}}\left(X\right)=\frac{1}{n-1}\overset{n}{\underset{t=2}{\sum }}\log P\left(x_{t}|x_{<t}\right)$$

Masked language models (MLMs) differ from autoregressive models in their inference behavior, as they are not inherently designed for sequential generation. To address this, Wang and Cho^21^ introduced the pseudo log-likelihood (PLL) approach, wherein each token in a sequence is masked individually to compute token-wise conditional probabilities. More recently, Gordon et al.^22^ proposed Single-Inference Pseudo Log-Likelihood, an efficient approximation that enables linear-time inference for BERT-style MLMs by exploiting training-time masking dynamics.

Rather than computing pseudo log-likelihood (PLL), we assessed relative changes in mean token-wise log-probabilities derived from unmasked logits for all MLM-based gLMs. This method, though lacking strict probabilistic interpretation, provides a scalable and effective proxy for quantifying model sensitivity to functional disruptions. The formula is as follows:

Given the *logits*_*t*_ at each position *t*, the probability of the correct token is computed as

3 $$P\left(x_{t}|X\right)=\frac{\exp\left({\mathrm{logits}}_{t,x_{t}}\right)}{\underset{v\in V}{\sum }\exp\left({\mathrm{logits}}_{t,v}\right)}$$

Then the log-likelihood for the entire sequence is computed over all positions:

4 $${\mathrm{LL}}_{\mathrm{MLM}}\left(X\right)=\frac{1}{n}\overset{n}{\underset{t=1}{\sum }}\log P\left(x_{t}|X\right)$$

where all positions contribute to the likelihood computation since no masking is performed during inference.

Because these scores arise from different training objectives and inference procedures, their absolute values should not be interpreted as being on a common probabilistic scale across model families. We therefore use them as model-specific sequence scores and restrict direct interpretation of score magnitude to within-model analyses; cross-model comparisons are based on derived metrics such as success rate and correlation patterns, rather than raw score magnitudes.

To assess significance between paired conditions (e.g., original vs mutant), we performed a one-sided paired permutation test. Given differences *d*_*i*_ = *x*_*i*_^*mutant*^ – *x*_*i*_^*original*^ for each pair *i*, we computed the observed mean *d̅*_obs_. Under the null hypothesis of no effect, signs of *d*_*i*_ are exchangeable. We generated *N* = 10,000 random permutations by independently sampling *s*_*i*_ ∈ {−1, 1} with equal probability and computing ${\mathrm{d̅}}^{\left(j\right)}=\frac{1}{n}\overset{n}{\underset{i=1}{\sum }}s_{i}d_{i}$ . The one-sided p-value was calculated as

5 $$p=\frac{1}{N}\overset{N}{\underset{j=1}{\sum }}I\left({\mathrm{d̅}}^{\left(j\right)}\leq {\mathrm{d̅}}_{\mathrm{obs}}\right)$$

for the alternative hypothesis that the mutant is smaller than the original. We further multiply this p-value by the number of total tests per model to adjust for multiple comparisons.

To further quantify the uncertainty of the paired effect size, we estimated 95% confidence intervals for the mean paired difference using nonparametric bootstrap resampling. For each paired comparison, we defined Δ_*i*_ = *x*_*i*_^*mutant*^ – *x*_*i*_^*original*^ and generated *B* = 10,000 bootstrap resamples by sampling with replacement from {Δ_*i*_}_*i* = 1_^*n*^, each of size *n* = 1,500. For each bootstrap resample *b*, we computed the mean difference

$${\mathrm{\Delta ̅}}^{\left(b\right)}=\frac{1}{n}\overset{n}{\underset{i=1}{\sum }}\Delta_{i}^{\left(b\right)}$$

The 95% confidence interval (CI) was then obtained from the 2.5th and 97.5th percentiles of the resulting bootstrap distribution of Δ̅, with CI width defined as the difference between the two values. This procedure was applied separately to each mutant type, data set, and model.

## Supplementary Material



## Data Availability

The source code used for gLM benchmarking and Nullsettes construction is publicly available at: https://github.com/cellethology/GLM-Nullsette-Benchmark. The expression cassette of Kosuri data set^11^ was obtained from https://www.addgene.org/47441/. The Lagator data set^9^ was downloaded from https://github.com/szarma/Thermoters. The deBoer data set^10^ was downloaded from https://www.ncbi.nlm.nih.gov/geo/query/acc.cgi?acc=GSE104878. The Zahm data set^12^ was downloaded from https://www.ncbi.nlm.nih.gov/geo/query/acc.cgi?acc=GSE271608. The parsed expression cassettes and Nullsettes data sets are available at https://github.com/cellethology/GLM-Nullsette-Benchmark.

## Notes

Supplementary Figures S1–S8: including promoter log-likelihood distributions, cumulative successful prediction counts across genomic language models, effect sizes of log-likelihood shifts, confidence-interval width comparisons, model-size comparisons, correlations between sequence features and model performance, and sensitivity analyses using different success-rate cutoffs; Supplementary Tables S1–S6: including prokaryotic and eukaryotic virtual mutant designs, representative genomic language model variants, model pretraining data sets and architectures, and expression cassette construction across MPRA data sets (PDF)
