# Supplementary material for: Evaluating DNA Function Understanding in Genomic Language Models Using Evolutionarily Implausible Sequences
Source: ACS Synth Biol. 2026 Jun 9;15(6):2256–63. doi: 10.1021/acssynbio.6c00024 (PMC13288862; doi:10.1021/acssynbio.6c00024)
Supplement: Supplementary file 1 [file sb6c00024_si_001.pdf]

Evaluating DNA Function Understanding in Genomic Language Models Using Evolutionarily Implausible Sequences

Shiyu Jiang[1,*], Xuyin Liu[1], and Zitong Jerry Wang[1,*]

[1]Center for Interdisciplinary Studies, School of Science, Westlake University, Hangzhou, China, 310030

[*]*Correspondence to* jiang.shiyu@ufl.edu and jerry@westlake.edu.cn

Supplementary Figures

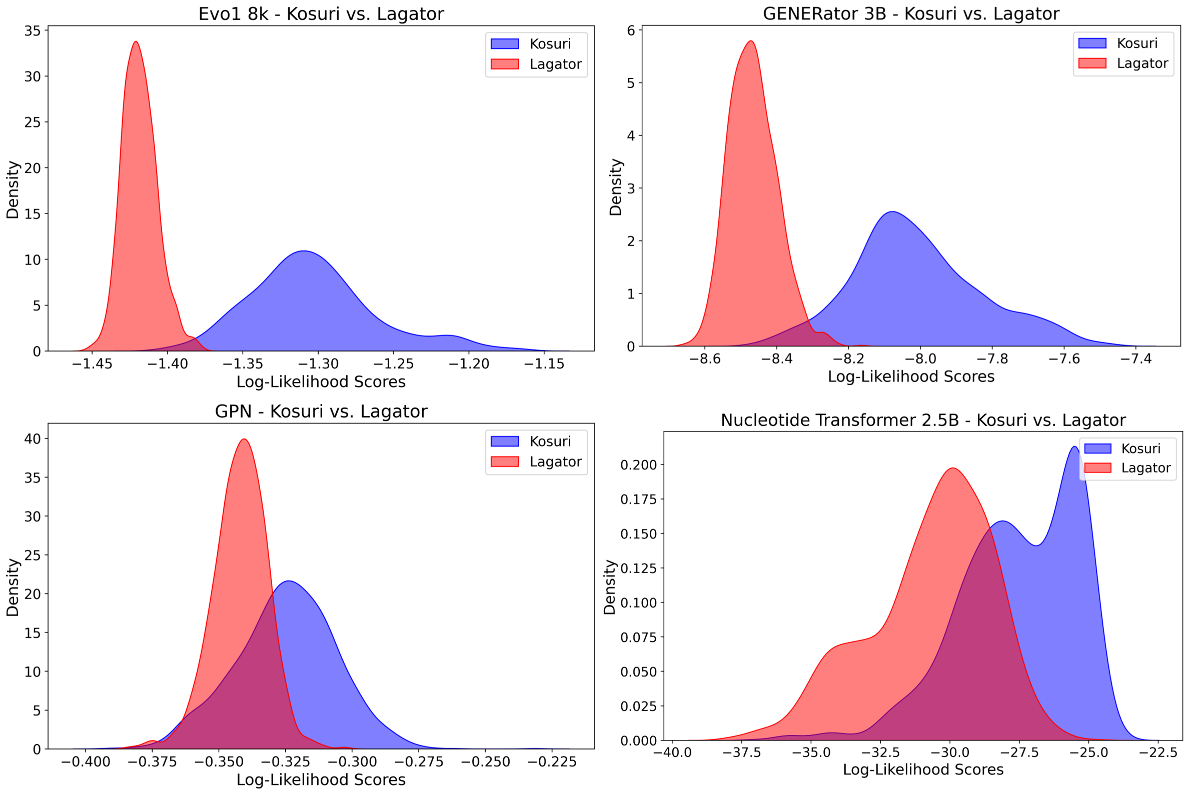

Figure S1: Comparison of promoter log-likelihood distributions.

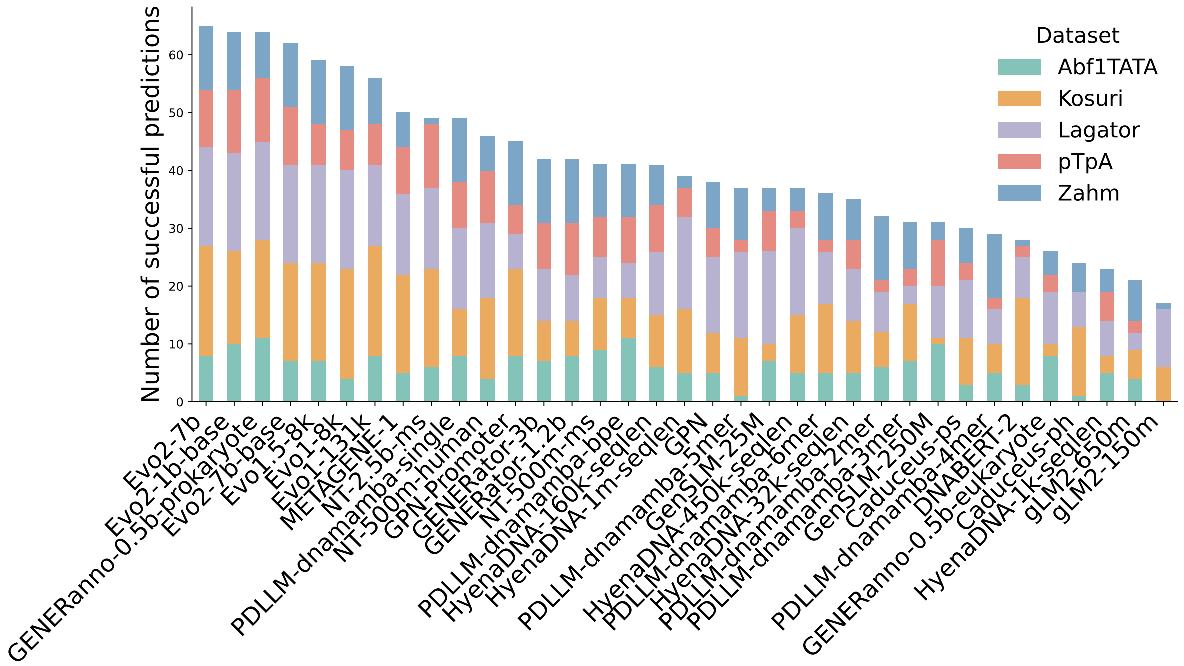

Figure S2: The number of cumulative successful predictions made by each model series across four datasets: Abf1TATA and pTpA (from deBoer), Zahm, Kosuri, and Lagator.

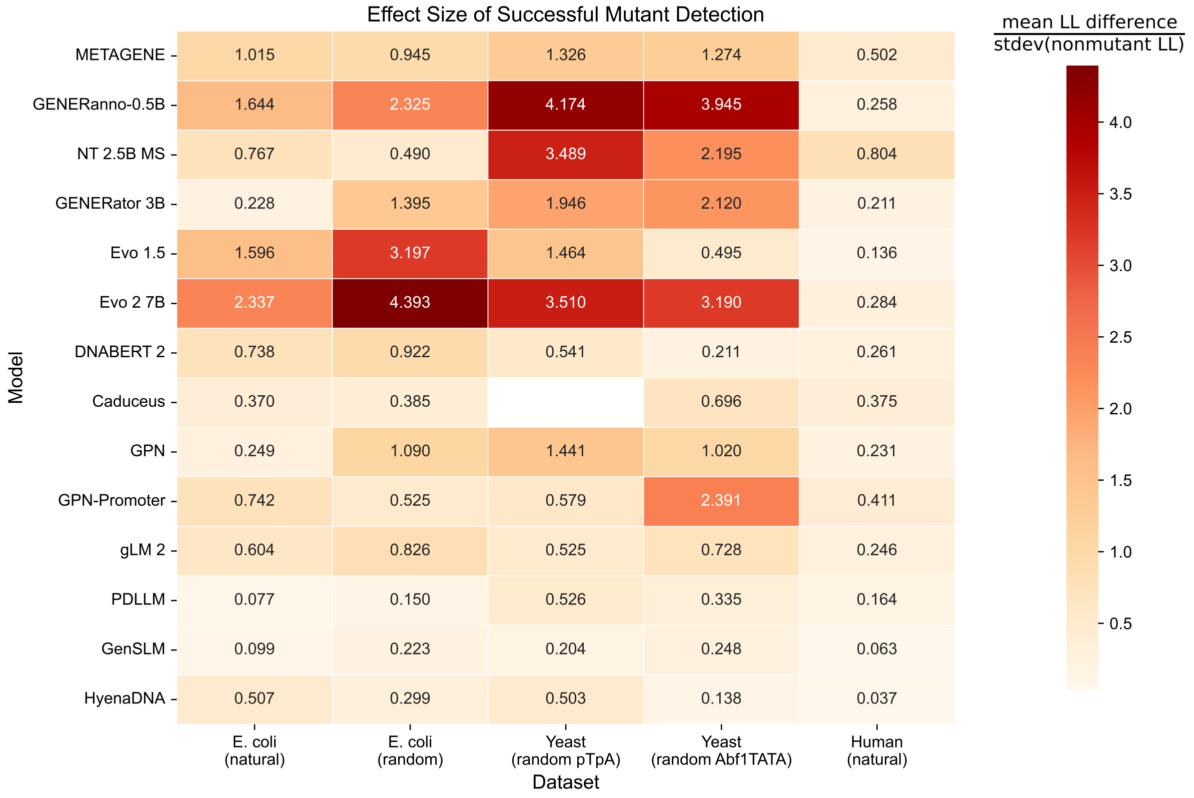

Figure S3: Effect sizes of LL shifts in successful mutant detection. Effect sizes were calculated as mean LL differences normalized by the standard deviation of nonmutant LL scores.

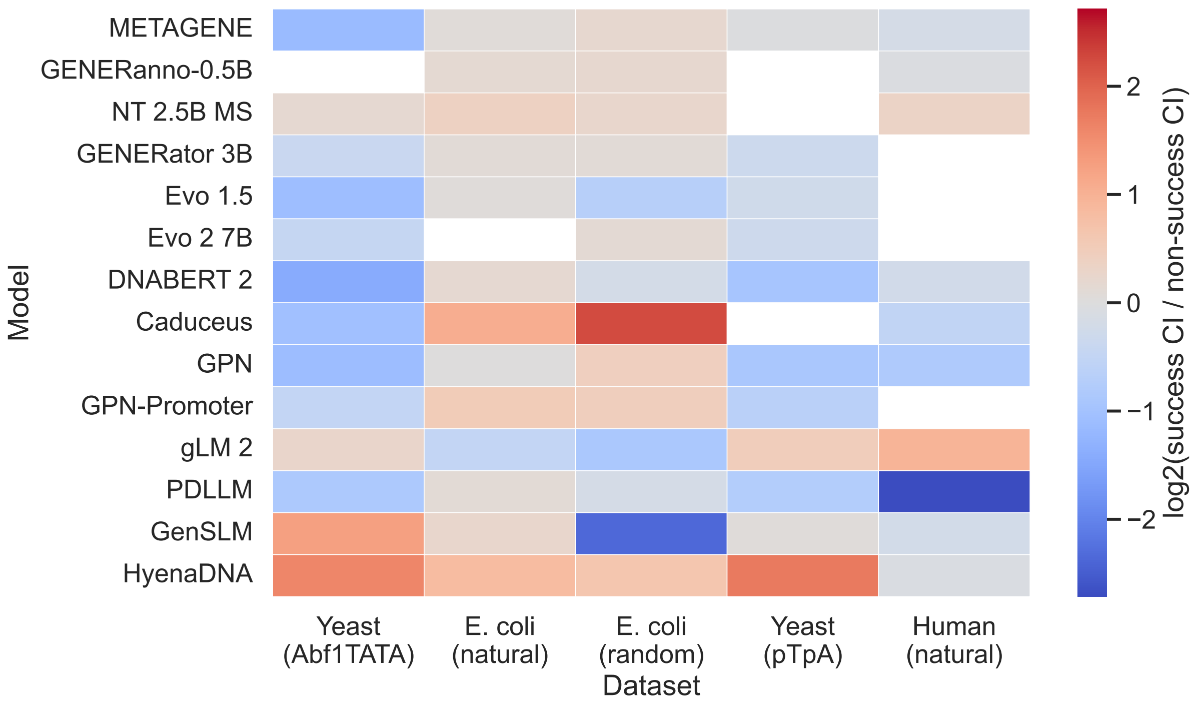

Figure S4: Log fold change in the 95% confidence-interval width for the LL difference, comparing successfully detected versus unsuccessfully detected mutants.

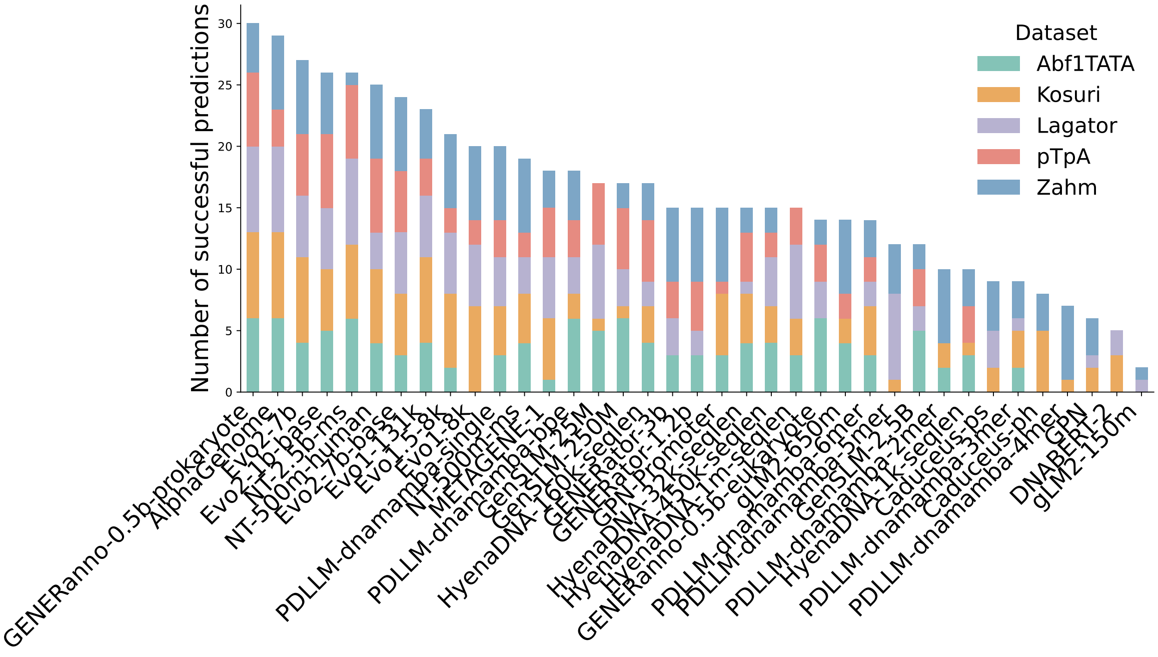

Figure S5: The number of cumulative successful predictions made by each model series, only on mutants with disrupted CDS transcription, including mutant 1, 7, 8, 9, 14, 16, 17 in Table S1 and mutant 1, 6, 7, 8, 9, 10 in Table S2

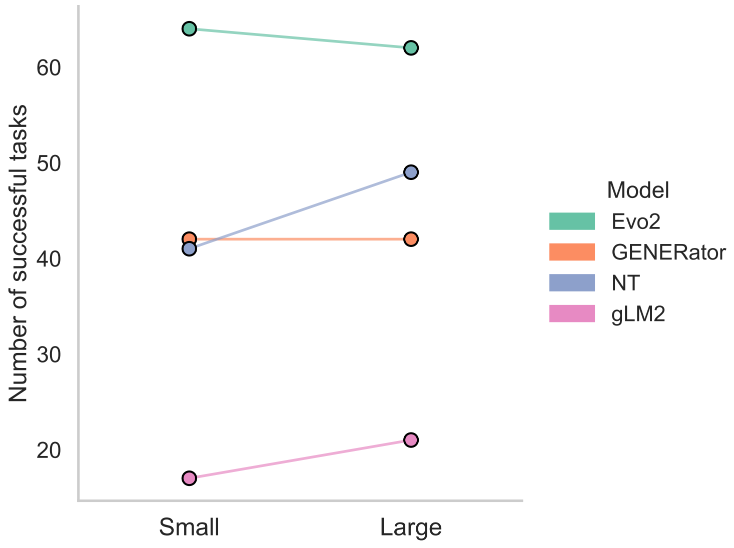

Figure S6: Number of Nullsette mutations successfully identified for gLMs with small and large variants, including Evo2 (1B vs. 7B), GENERator (1.2B vs. 3B), NT (500M vs. 2.5B, multispecies), and gLM2 (150M vs. 650M).

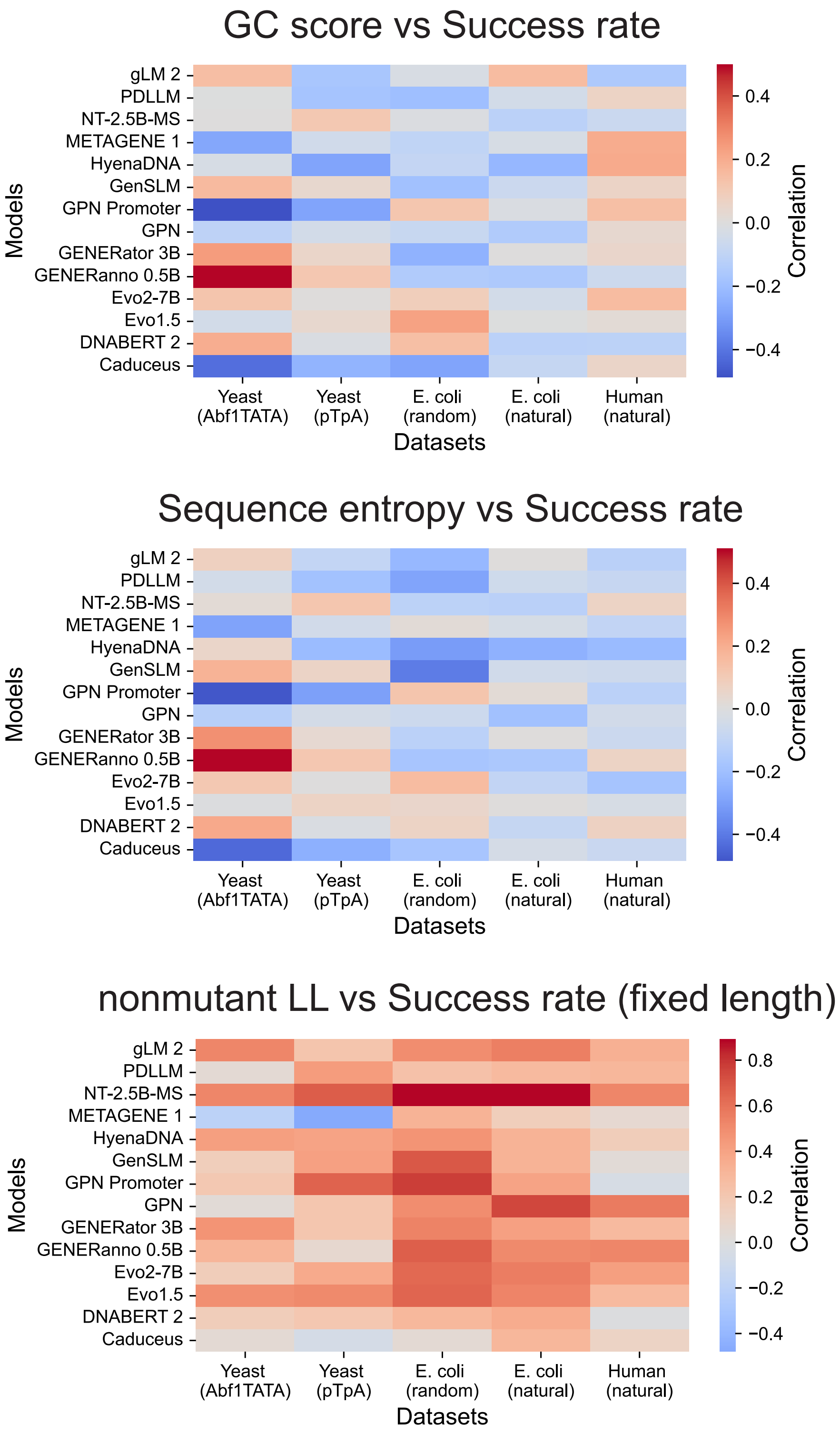

Figure S7: Correlation between sequence features and model performance on Nullsettes. GC content refers to the proportion of G's and C's in the nonmutant, sequence entropy is the standard Shannon entropy of the nonmutant sequence. For the last heatmap, we subset for sequences with length equal to the modal length for each dataset.

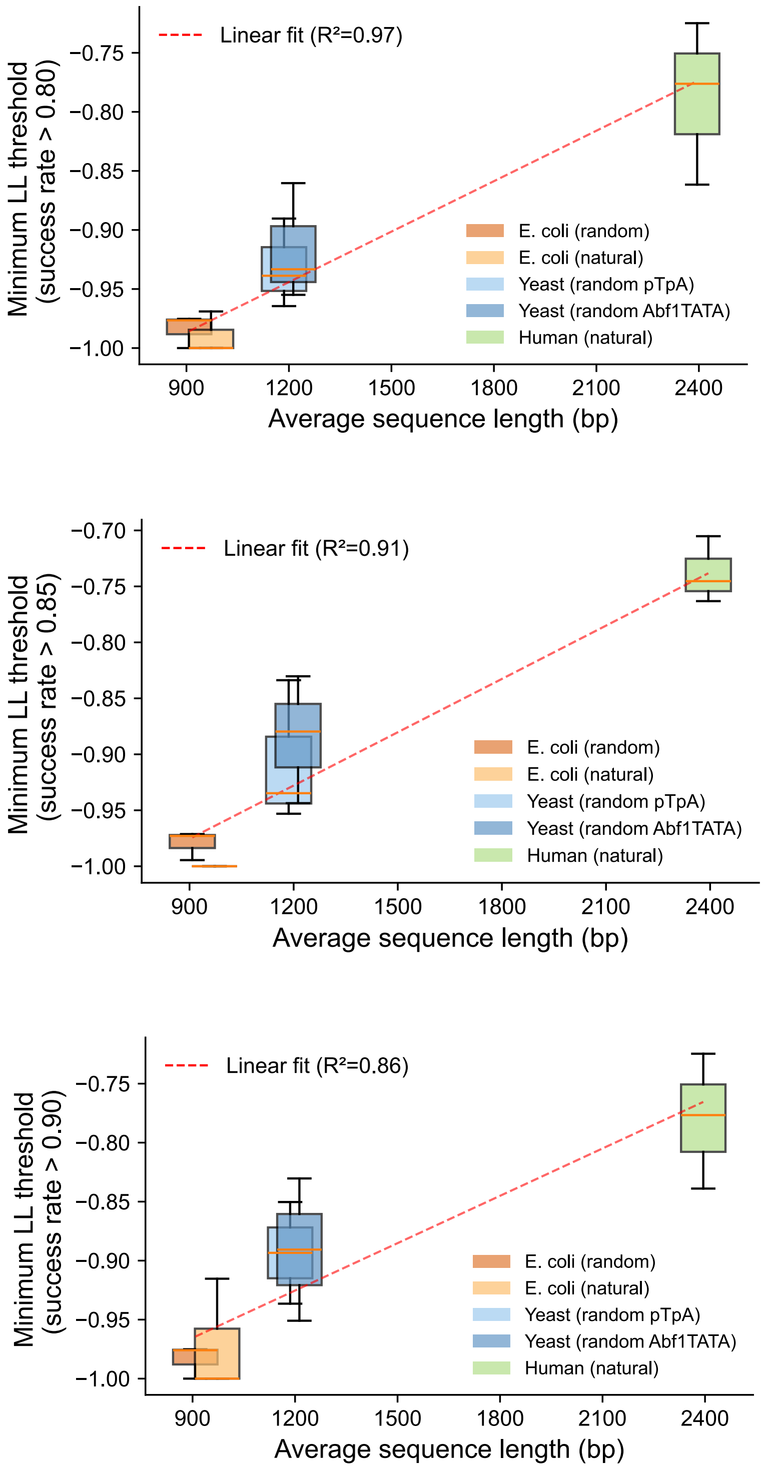

Figure S8: Different versions of Figure 2C with different success rate cutoffs.

Supplementary Tables

Mutant ID	Description
Translocation-1	CDS - Promoter - RBS - Start codon - Stop codon - Terminator
Translocation-2	Promoter - CDS - RBS - Start codon - Stop codon - Terminator
Translocation-3	Promoter - RBS - CDS - Start codon - Stop codon - Terminator
Translocation-4	Promoter - RBS - CDS - Stop codon - Start codon - Terminator
Translocation-5	Promoter - RBS - CDS - Stop codon - Terminator - Start codon
Translocation-6	Promoter - RBS - Start codon - Stop codon - CDS - Terminator
Translocation-7	Promoter - RBS - Start codon - Stop codon - Terminator - CDS
Translocation-8	Promoter - RBS - Start codon - Terminator - CDS - Stop codon
Translocation-9	Promoter - RBS - Terminator - Start codon - CDS - Stop codon
Translocation-10	Promoter - Start codon - CDS - RBS - Stop codon - Terminator
Translocation-11	Promoter - Start codon - CDS - Stop codon - RBS - Terminator
Translocation-12	Promoter - Start codon - CDS - Stop codon - Terminator - RBS
Translocation-13	Promoter - Start codon - RBS - CDS - Stop codon - Terminator
Translocation-14	Promoter - Terminator - RBS - Start codon - CDS - Stop codon
Translocation-15	RBS - Promoter - Start codon - CDS - Stop codon - Terminator
Translocation-16	RBS - Start codon - CDS - Promoter - Stop codon - Terminator
Translocation-17	RBS - Start codon - CDS - Stop codon - Promoter - Terminator
Translocation-18	RBS - Start codon - Promoter - CDS - Stop codon - Terminator
Translocation-19	Start codon - Promoter - RBS - CDS - Stop codon - Terminator

Table S1: **Prokaryotic virtual mutant cases.** Translocation-3,4,5 cannot be compensated by a later start codon as there is no other in-frame start codon.

Mutant ID	Description
Translocation-1	CDS - Promoter - Start codon - Stop codon - Terminator
Translocation-2	Promoter - CDS - Start codon - Stop codon - Terminator
Translocation-3	Promoter - CDS - Stop codon - Start codon - Terminator
Translocation-4	Promoter - CDS - Stop codon - Terminator - Start codon
Translocation-5	Promoter - Start codon - Stop codon - CDS - Terminator
Translocation-6	Promoter - Start codon - Stop codon - Terminator - CDS
Translocation-7	Promoter - Start codon - Terminator - CDS - Stop codon
Translocation-8	Promoter - Terminator - Start codon - CDS - Stop codon
Translocation-9	Start codon - CDS - Promoter - Stop codon - Terminator
Translocation-10	Start codon - CDS - Stop codon - Promoter - Terminator
Translocation-11	Start codon - Promoter - CDS - Stop codon - Terminator

Table S2: **Eukaryotic virtual mutant cases.** Translocation-2,3,4 cannot be compensated by a later start codon as there is no other in-frame start codon.

Model series	Representative variant
METAGENE1 [1]	METAGENE-1 (METAGENE1)
Nucleotide Transformer [2]	nucleotide-transformer-2.5b-multi-species (NT-2.5B-NT)
GENERator [3]	GENERator-eukaryote-3b-base (GENERator-3B)
GENERanno [4]	GENERanno-prokaryote-0.5b-base (GENERanno-0.5B)
Evo1 [5, 6]	evo-1.5-8k-base (Evo1.5)
Evo2 [7]	evo2_7b (Evo2-7B)
DNABERT2 [8]	DNABERT-2-117M (DNABERT2)
Caduceus [9]	caduceus-ph_seqlen-131k_d_model-256_n_layer-16 (Caduceus)
GPN [10]	gpn-brassicales (GPN)
GPN-Promoter [11]	gpn-animal-promoter (GPN-Promoter)
gLM2 [12]	gLM2_650M (gLM2)
PDLLM [13]	PDLLM-dnamamba-3mer (PDLLM)
GenSLM [14]	GenSLM-2.5B (GenSLM)
HyenaDNA [15]	HyenaDNA-32k-seqlen (HyenaDNA)

Table S3: **Representative model variant.** For each model series, we list a representative variant used in major benchmarking. The official model identifier is listed, followed in parentheses by the shorthand name used throughout the main text. For example, the variant "nucleotide-transformer-2.5b-multi-species" is referred to as "NT-2.5B-NT" in the text.

Model name	Pretraining dataset	Pretraining method	Tokenization	Architecture	Input length
Evo1 8k [5]	OpenGenome: Prokaryotic whole-genomes dataset (300B tokens).	CLM	Single-nucleotide tokens	StripedHyena, 7B params	Pretrained with 8,192 context
Evo1 131k [5]	OpenGenome: Prokaryotic whole-genomes dataset (300B tokens).	CLM	Single-nucleotide tokens	StripedHyena, 7B params	Pretrained with 131,072 context using Evo1 8k as the base model
Evo1.5 [6]	50% increase in training data compared to Evo1	CLM	Single-nucleotide tokens	StripedHyena, 7B params	Pretrained with 8,192 context using Evo1 8k as the base model
Evo2 1B base [7]	OpenGenome2: a dataset containing 8.8 trillion tokens from all domains of life.	CLM	Single-nucleotide tokens	StripedHyena 2, 1B params	Pretrained with 8192 context length
Evo2 7B base [7]	OpenGenome2: a dataset containing 8.8 trillion tokens from all domains of life.	CLM	Single-nucleotide tokens	StripedHyena 2, 7B params	Pretrained with 8192 context length
Evo2 7B [7]	OpenGenome2: a dataset containing 8.8 trillion tokens from all domains of life.	CLM	Single-nucleotide tokens	StripedHyena 2, 7B params	Pretrained with 1M context using Evo2 7b base as the base model
GENERator eukaryote 3B [3]	Eukaryotic genomes (386B bp), plants, fungi, protozoa, mammalian, vertebrate, invertebrate.	CLM	6-mer	Llama-based decoder, 3B params	Pretrained with a context length of 98k bp
GENERator eukaryote 1.2B [3]	Eukaryotic genomes (386B bp), plants, fungi, protozoa, mammalian, vertebrate, invertebrate.	CLM	6-mer	Llama-based decoder, 1.2B params	Pretrained with a context length of 98k bp
METAGENE-1 [1]	1.5T base pairs of DNA and RNA sequences from human wastewater samples.	CLM	Byte-pair encoding	Llama-2, 7B params	Pretrained with 512 sequence length
PDLLM-DNAMamba [13]	Plant reference genomes	CLM	6-mer/5-mer/4-mer/3-mer/2-mer/single/BPE	SSM, 130M params	Pretrained max token length is 512
GenSLM [14]	BV-BRC/PATRIC prokaryotic gene sequences (110M sequences)	CLM	Codon-level (3-mer)	GPT-based decoder-only Transformer, 25M–25B params	Pretrained max token length is 2048; 25M/250M variants were further trained with 10240 context on full SARS-CoV-2 genomes
HyenaDNA [15]	Human reference genome (hg38)	CLM	Single-nucleotide tokens	Decoder-only Hyena, 0.44M–6.6M params	Pretrained max token length is 1M

Table S4: Causal language modeling (CLM) based genomic language models

**Model name**	**Pretraining dataset**	**Pretraining method**	**Tokenization**	**Architecture**	**Input length**
Nucleotide Transformer 2.5B multispecies [2]	850 whole genomes from NCBI, plants and viruses are not included, resulting in a total of 174B nucleotides, i.e. roughly 29B tokens.	MLM	6-mers	Transformer, 2.5B params	1000 bp
Nucleotide Transformer 500M multispecies [2]	850 whole genomes from NCBI, plants and viruses are not included, resulting in a total of 174B nucleotides, i.e. roughly 29B tokens.	MLM	6-mers	Transformer, 500M params	1000 bp
Nucleotide Transformer 500M Human ref [2]	GRCh38 human reference genome, resulting in 3B nucleotides, i.e. roughly 500M 6-mer tokens.	MLM	6-mers	Transformer, 500M params	1000 bp
GENERanno prokaryote 0.5B base [4]	715 billion base pairs of prokaryotic DNA	MLM	single-nucleotide	Transformer encoder, 500M params	8k bp
GENERanno eukaryote 0.5B base [4]	386 billion base pairs of eukaryotic DNA	MLM	single-nucleotide	Transformer encoder, 500M params	8k bp
Caduceus-ph [9]	Human reference genome (35M tokens / nucleotide base pairs).	MLM	Single-nucleotide tokens	BiMamba, 7.7M params	Pretrained with 131,072 sequence length (reverse complement augmentation)
Caduceus-ps [9]	Human reference genome (35M tokens / nucleotide base pairs).	MLM	Single-nucleotide tokens	BiMamba, 7.7M params	Pretrained with 131,072 sequence length (No reverse complement augmentation)
GPN [10]	Brassicales reference genome from NCBI Genome. Took the union of exons (with a small intronic flank), promoters (1,000 bp upstream of transcription start sites) as well as an equivalent amount of random windows from the whole genome.	MLM	Single-nucleotide tokens	CNN	512 bp
GPN-Promoter [11]	Animal promoter, genomes of 434 animal species.	MLM	Single-nucleotide tokens	ByteNet, 152M	Pretrained on 512 bp sequences centered at TSSs of protein-coding genes
DNABERT2 [8]	Human genome dataset (2.75B nucleotide bases) + Multispecies genome dataset (from 135 species, spread across 6 cetogories). The dataset includes 32.49B nucleotides bases, excluding all sequences with N and retaining only ATCG.	MLM	Byte-pair encoding	BERT, 117M params	Pretrained on 700 bp length sequences
gLM2 [12]	OMG: encodes genomic scaffolds with both amino-acid (CDS) and DNA tokens (315B tokens).	MLM	Char-level tokens (DNA: lowercase, AA: uppercase)	Transformer, 650M params / 150M params	Pretrained with a 4096 token context window

Table S5: Masked language modeling (MLM) based genomic language models

Dataset	Type	Expression cassette construction	Description
deBoer-pTpA	Hybrid	distal promoter – promoter – proximal promoter – linker – kozak – ATG start codon – yeGFP (CDS) – TAA stop codon – linker – ADH1 terminator	In the deBoer-pTpA cassette, only the “promoter” segment is randomized, while all other components are fixed. This library is classified as hybrid because the inserted promoters are synthetic sequences built on a poly-T/poly-A architecture, combining randomized segments with promoter motif.
deBoer-Abf1TATA	Hybrid	distal promoter – promoter – proximal promoter – linker – kozak – ATG start codon – yeGFP (CDS) – TAA stop codon – linker – ADH1 terminator	In the deBoer-Abf1TATA cassette, only the “promoter” segment is variable, while all other components are fixed. This library is classified as hybrid because the variable promoters are designed around conserved regulatory motifs, including an Abf1 binding site and a canonical TATA box, thereby combining natural cis-regulatory elements with randomized sequence context.
Kosuri	Natural	promoter – rbs – ATG start codon – superfolder GFP (CDS) – TAA stop codon – linker – rrnB T1 terminator	In the Kosuri cassette, the variable region comprises the paired “promoter – rbs” segment, whereas the remainder of the cassette is fixed. This dataset is treated as natural because it uses combinatorial promoter and ribosome-binding-site assemblies rather than fully randomized regulatory sequences, enabling the dissection of transcriptional and translational contributions to gene expression.
Legator	Randomized	promoter – linker – rbs – linker – ATG start codon – YFP (CDS) – TAG stop codon – linker – rrnB T1 terminator	In the Legator cassette, only the “promoter” segment is variable, while all other components are fixed. The variable region consists of a large-scale library of randomized promoter sequences.
Zahm-minCMV	Natural	TRE – minCMV promoter – linker – kozak – ATG start codon – luciferase (CDS) – TAA stop codon – linker – bGH poly(A) terminator	Across the Zahm constructs, the variable component is the “TRE” (transcriptional response element), whereas the minimal promoter (“minCMV”, “minProm”, or “minTK”) is fixed within each cassette background and all downstream components are fixed. These datasets are treated as natural in our classification because they are built from libraries of transcriptional response elements associated with human and mouse transcription factors and paired with defined minimal promoters.
Zahm-minProm	Natural	TRE – minProm promoter – linker – kozak – ATG start codon – luciferase (CDS) – TAA stop codon – linker – bGH poly(A) terminator	
Zahm-minTK	Natural	TRE – minTK promoter – linker – kozak – ATG start codon – luciferase (CDS) – TAA stop codon – linker – bGH poly(A) terminator	

Table S6: **Expression cassette construction across datasets.** For each dataset, we summarize the expression cassette architecture, classify the sequence source as randomized or natural, and indicate which sequence segment varies within the corresponding cassette design. In Nullsettes construction, the translocated promoter unit is defined according to the dataset-specific variable segment composition.

References

[1] Ollie Liu, Sami Jaghouar, Johannes Hagemann, Shangshang Wang, Jason Wiemels, Jeff Kaufman, and Willie Neiswanger. Metagene-1: Metagenomic foundation model for pandemic monitoring. arXiv preprint arXiv:2501.02045, 2025.

[2] Hugo Dalla-Torre, Liam Gonzalez, Javier Mendoza-Revilla, Nicolas Lopez Carranza, Adam Henryk Grzywaczewski, Francesco Oteri, Christian Dallago, Evan Trop, Bernardo P de Almeida, Hassan Sirelkhatim, et al. Nucleotide transformer: building and evaluating robust foundation models for human genomics. Nature Methods, pages 1–11, 2024.

[3] Wei Wu, Qiuyi Li, Mingyang Li, Kun Fu, Fuli Feng, Jieping Ye, Hui Xiong, and Zheng Wang. Generator: A long-context generative genomic foundation model. arXiv preprint arXiv:2502.07272, 2025.

[4] Qiuyi Li, Wei Wu, Yiheng Zhu, Fuli Feng, Jieping Ye, and Zheng Wang. Generanno: A genomic foundation model for metagenomic annotation. bioRxiv, pages 2025–06, 2025.

[5] Eric Nguyen, Michael Poli, Matthew G Durrant, Brian Kang, Dhruva Katrekar, David B Li, Liam J Bartie, Armin W Thomas, Samuel H King, Garyk Brixi, et al. Sequence modeling and design from molecular to genome scale with evo. Science, 386(6723):eado9336, 2024.

[6] Aditi T Merchant, Samuel H King, Eric Nguyen, and Brian L Hie. Semantic mining of functional de novo genes from a genomic language model. bioRxiv, pages 2024–12, 2024.

[7] Garyk Brixi, Matthew G Durrant, Jerome Ku, Michael Poli, Greg Brockman, Daniel Chang, Gabriel A Gonzalez, Samuel H King, David B Li, Aditi T Merchant, et al. Genome modeling and design across all domains of life with evo 2. bioRxiv, pages 2025–02, 2025.

[8] Zhihan Zhou, Yanrong Ji, Weijian Li, Pratik Dutta, Ramana Davuluri, and Han Liu. Dnabert-2: Efficient foundation model and benchmark for multi-species genome. arXiv preprint arXiv:2306.15006, 2023.

[9] Yair Schiff, Chia-Hsiang Kao, Aaron Gokaslan, Tri Dao, Albert Gu, and Volodymyr Kuleshov. Caduceus: Bi-directional equivariant long-range dna sequence modeling. arXiv preprint arXiv:2403.03234, 2024.

[10] Gonzalo Benegas, Sanjit Singh Batra, and Yun S Song. Dna language models are powerful predictors of genome-wide variant effects. Proceedings of the National Academy of Sciences, 120(44):e2311219120, 2023.

[11] Gonzalo Benegas, Gökcen Eraslan, and Yun S Song. Benchmarking dna sequence models for causal regulatory variant prediction in human genetics. bioRxiv, pages 2025–02, 2025.

[12] Andre Cornman, Jacob West-Roberts, Antonio Pedro Camargo, Simon Roux, Martin Beracochea, Milot Mirdita, Sergey Ovchinnikov, and Yunha Hwang. The omg dataset: An open metagenomic corpus for mixed-modality genomic language modeling. bioRxiv, pages 2024–08, 2024.

[13] Guanqing Liu, Long Chen, Yuechao Wu, Yangshuo Han, Yu Bao, and Tao Zhang. Pdllms: A group of tailored dna large language models for analyzing plant genomes. Molecular Plant, 18(2):175–178, 2025.

[14] Maxim Zvyagin, Alexander Brace, Kyle Hippe, Yuntian Deng, Bin Zhang, Cindy Orozco Bohorquez, Austin Clyde, Bharat Kale, Danilo Perez-Rivera, Heng Ma, et al. Genslms: Genome-scale language models reveal sars-cov-2 evolutionary dynamics. The International Journal of High Performance Computing Applications, 37(6):683–705, 2023.

[15] Eric Nguyen, Michael Poli, Marjan Faizi, Armin Thomas, Michael Wornow, Callum Birch-Sykes, Stefano Massaroli, Aman Patel, Clayton Rabideau, Yoshua Bengio, et al. Hyenadna: Long-range genomic sequence modeling at single nucleotide resolution. Advances in neural information processing systems, 36:43177–43201, 2023.
